# RETRACTION: MicroRNA‐342 Promotes the Malignant‐Like Phenotype of Endometrial Stromal Cells via Regulation of Annexin A2

**DOI:** 10.1155/ancp/9820195

**Published:** 2026-09-24

**Authors:** Analytical Cellular Pathology

RETRACTION: D. Sun, Y. Wang, L. Wang, and X. Guo, “MicroRNA‐342 Promotes the Malignant‐Like Phenotype of Endometrial Stromal Cells via Regulation of Annexin A2,” *Analytical Cellular Pathology* 2021, no. 1 (2021): https://doi.org/10.1155/2021/1328682.

The above article, published online on 17 May 2021 in Wiley Online Library (wileyonlinelibrary.com), has been retracted by agreement between the journal Editor‐in‐Chief, Dimitrios Karamichos, and John Wiley & Sons Ltd. The retraction has been agreed after an investigation in to concerns raised by *Hoya camphorifolia* and *Indigofera tanganyikensis* on PubPeer [1].

Specifically:

• Figure 4d, second panel, shares unexpected similarities with Figure 3c, third panel, in [2];
• The flow cytometry data presented in Figures 2e, 4e and 5h show unusual distributions, and appear to be inaccurately measured.

The authors initially requested a correction to Figures 2, 4 and 5. However, several attempts by the journal to contact the authors regarding the abovementioned concerns have not resulted in a reply. As such, the results reported in this paper cannot be considered reliable.
